# Analysis of the complete plastidial genome of the newly highland papaya *Vasconcellea carvalhoae* (Caricaceae) from Peru

**DOI:** 10.1080/23802359.2022.2135407

**Published:** 2022-10-27

**Authors:** Daniel Tineo, Danilo E. Bustamante, Martha S. Calderon

**Affiliations:** a Instituto de Investigación para el Desarrollo Sustentable de Ceja de Selva (INDES-CES), Universidad Nacional Toribio Rodríguez de Mendoza, Chachapoyas, Peru; b Instituto de Investigación en Ingeniería Ambiental (IIIA), Facultad de Ingeniería Civil y Ambiental (FICIAM), Universidad Nacional Toribio Rodríguez de Mendoza, Chachapoyas, Peru

**Keywords:** Amazonas region, Brassicales, highland papaya, Peru, plastid genome, Vasconcellea

## Abstract

Especially in South American Andean communities, *Vasconcellea carvalhoae* D. Tineo & D.E. Bustamante 2020 is a significant highland papaya with agronomic promise. High-throughput sequencing of the holotype specimen of *V. carvalhoae* from Peru (KUELAP227) resulted in the assembly of its complete plastid genome (GenBank accession number ON764441). The plastid genome of this highland papaya is 158,723 bp and contains 130 genes. This plastid genome is similar in length, content, and organization to other members of Caricaceae, except for the absence of the pseudogene infA. Phylogenetic analyses of *V. carvalhoae* support its sistership to *V. pubescens*.

## Graphical Abstract

**Figure:**
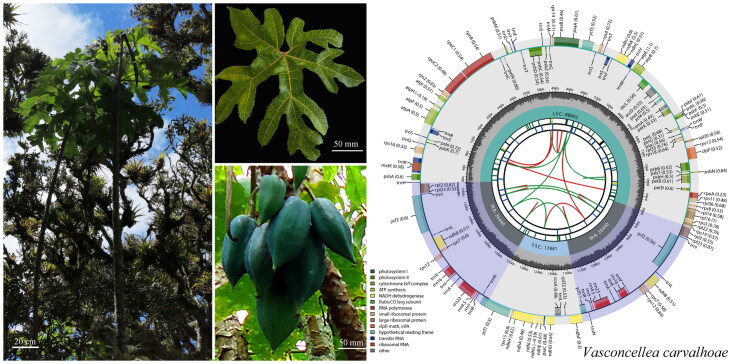

*Vasconcellea* is the most speciose genus in Caricaceae and traditionally known as highland papayas (Carvalho 2013, ). These species offer a variety of beneficial traits that point to their potential as an agricultural crop, particularly in Andean communities. These beneficial traits include disease resistance, cold tolerance, high latex enzymatic activity, and high protein and vitamin contents (Scheldeman et al. 2011). *Vasconcellea carvalhoae* is a newly described species of mountain papayas distributed in the wild in montane areas at 2236 m elevation in northeastern Peru (Tineo et al. 2020). This species is characterized by being dioecious trees to 4 m tall. *V. carvalhoae* exhibit morphological similarities to *V. pubescens.* These species are growing in sympatry; however, *V. carvalhoae* is distinguished by their elongated ovoid berry, lack of pubescence, and 0.36% of genetic divergence for the ITS marker (Tineo et al. 2020). To better understand the evolutionary relationship of *V. carvalhoae*, the complete plastid genome of the holotype of *V. carvalhoae* was characterized.

The holotype of *V. carvalhoae* (Figure 1) was deposited at the herbarium of Universidad Nacional Toribio Rodríguez de Mendoza (KUELAP, https://www.untrm.edu.pe, Curator Eli Pariente, email: eli.pariente@untrm.edu.pe) under the voucher number KUELAP227 (collected by Daniel Tineo from Pomacochas, Peruvian Amazonas region; 5°49′45″S, 77°58′12″W).

**Figure 1.**
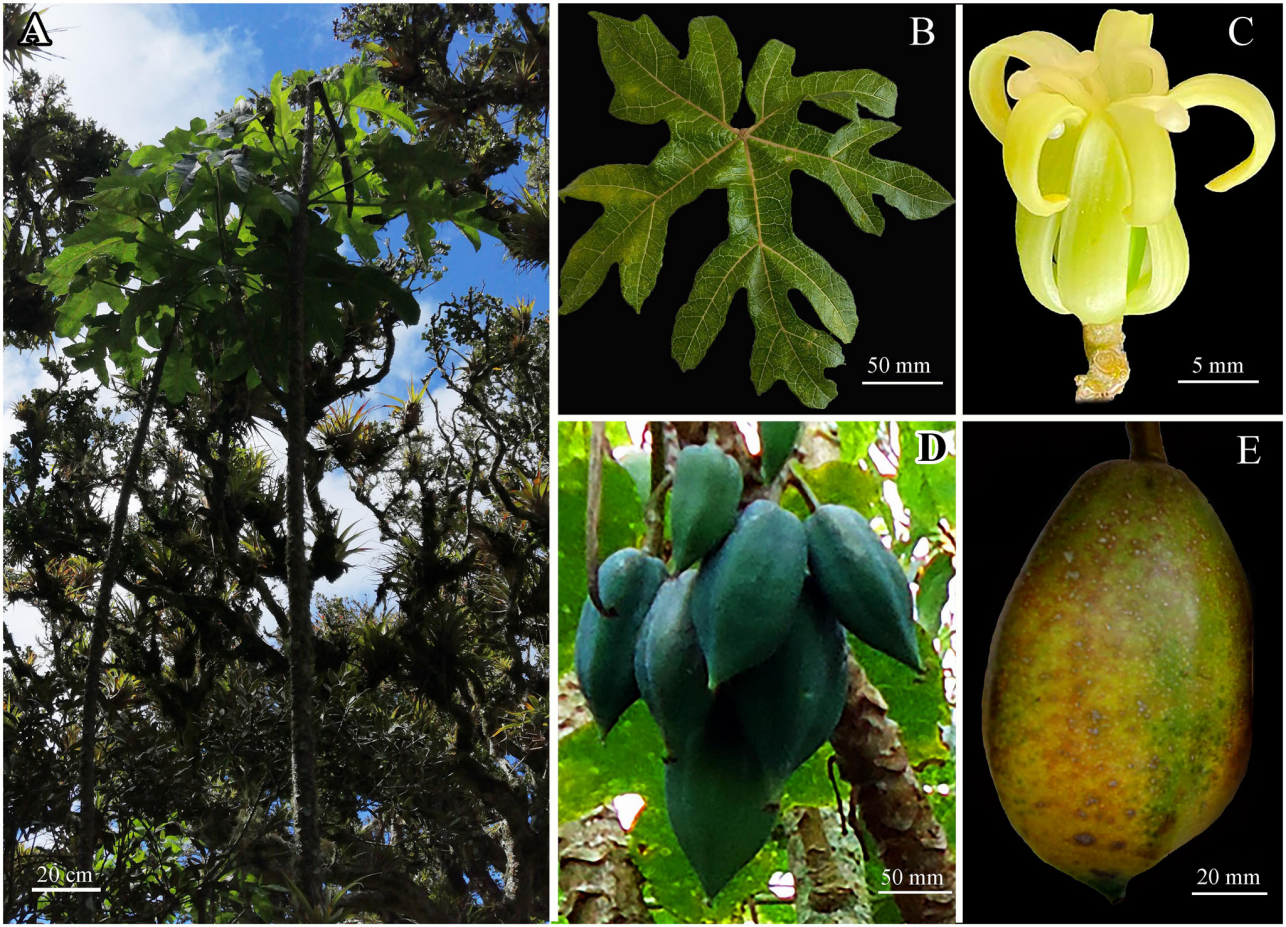
Morphology of *Vasconcellea carvalhoae* (KUELAP227). (A) Habit. (B) Palmately compound leaf. (C) Female flowers. (D) Immature fruits. (G) Mature fruit. All images have been obtained from Daniel Tineo from the field and Herbarium Kuelap.

DNA genomic was extracted from *V. carvalhoae* (specimen voucher number KUELAP227) using the kit NucleoSpin (Macherey-Nagel, Düren, Germany) following the manufacturer’s instructions. Genomic DNA was commercially sequenced by myGenomics, LLC (Alpharetta, GA). Genomic DNA was fragmented and ligated to unique adapters using Swift 2S Turbo DNA library prep kit (Swift Bioscience, Ann Harbor, MI). The libraries were then sequenced on NextSeq 500 (Illumina, San Diego, CA) according to the standard operation. Paired-end 150 nucleotide (nt) reads were generated. The genome was assembled using default de novo settings in MEGAHIT (Li et al. 2015) and Geneious Prime (Biomatters Ltd., Auckland, New Zealand) to close gaps. The genes were annotated manually using blastx, NCBI ORFfinder, and tRNAscan-SE 2.0 (Lowe and Chan 2016). The *V. carvalhoae* plastid genome was aligned to other plastomes using MAFFT (Katoh and Standley 2013). The phylogenetic analysis was performed with RAxML-NG (Kozlov et al. 2019) using the GTR + gamma model and 1000 bootstraps (Bustamante et al. 2020). The tree was visualized with TreeDyn 198.3 at Phylogeny.fr (Dereeper et al. 2008).

The plastid genome of *V. carvalhoae* is 158,723 bp in length and contains 130 genes (Figure 2). It is A + T rich (63.02%), and includes 25 ribosomal proteins, 37 tRNA (trnA, trnE, trnM, trnN, and trnT occur in duplicates; trnI, trnL, trnR, and trnV occur in triplicates; and trnS occurs in quintuplet), 22 photosystem I and II, six ycf, seven cytochrome b/f complex, six ATP synthase, four RNA polymerase, eight rRNA, and 15 other genes. Additionally, 12 cis-splicing and one trans-splicing genes were identified (Figures S1 and S2). Fifty-two of the 130 genes are transcribed on the forward strand and the remaining 78 are coded on the reverse strand.

**Figure 2.**
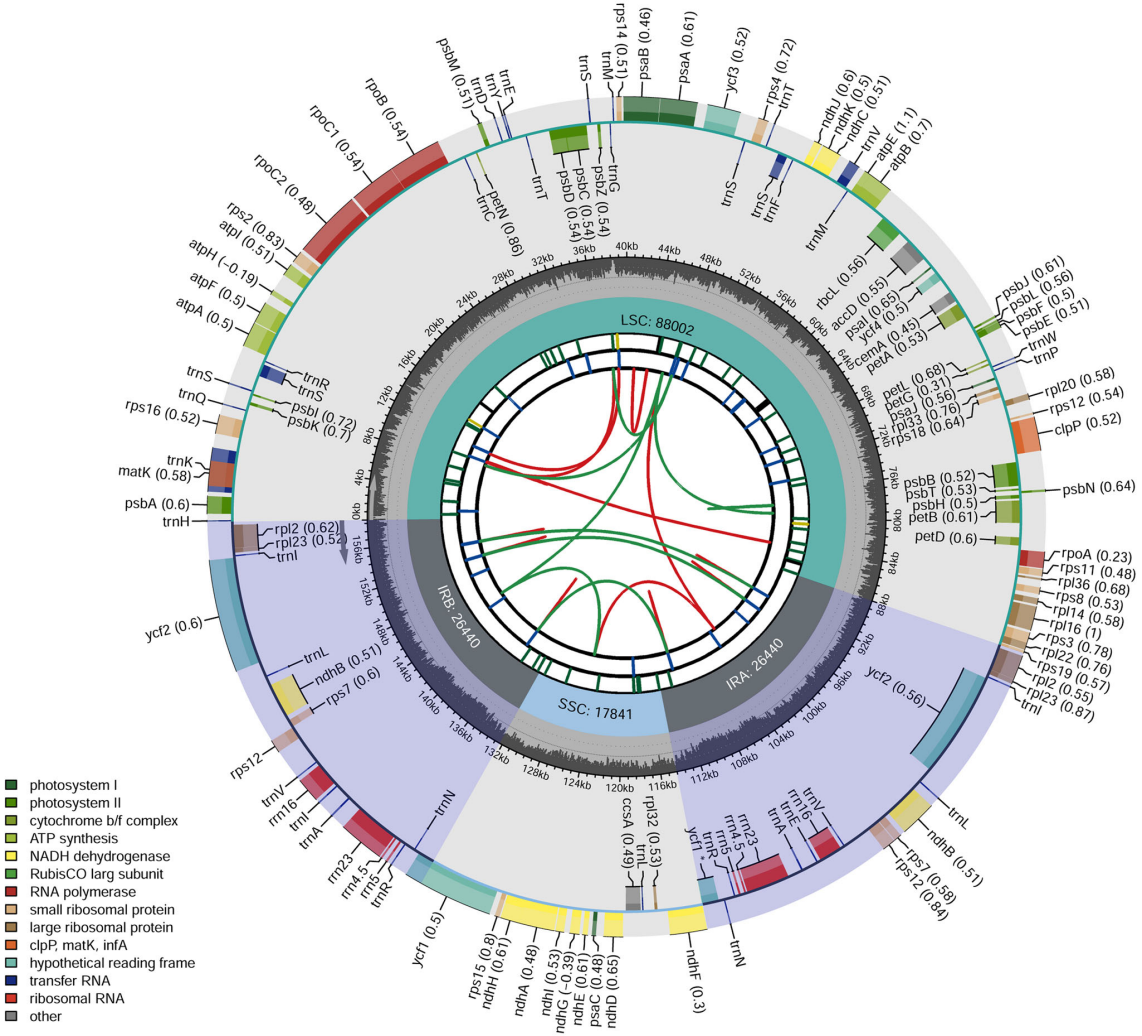
Schematic map of overall features of the chloroplast genome of *Vasconcellea carvalhoae*. The map contains six tracks in default. From the center outward, the first track shows the dispersed repeats connected with arcs. The second track shows the long tandem repeats as short bars. The third track shows the short tandem repeats or microsatellite sequences as short bars. The small single-copy (SSC), inverted repeat (IRa and IRb), and large single-copy (LSC) regions are shown on the fourth track. The GC content along the genome is plotted on the fifth track. The genes are shown on the sixth track. The optional codon usage bias is displayed in the parenthesis after the gene name. Genes are coded by their functional classification. The transcription directions for the inner and outer genes are clockwise and anticlockwise, respectively. The functional classification of the genes is shown in the bottom left corner.

The plastid genome of *V. carvalhoae* is highly conserved in length, content, and organization to other genera currently assigned to the Caricaceae, except for the absence of the pseudogen infA (Table 1), which is one of the essential components for the initiation of protein synthesis (The UniProt Consortium 2021). The plastid genome of *V. carvalhoae* is slightly larger than *V. pubescens* (158,712 bp; Lin et al. 2020) but shorter than *Carica papaya* plastome (160,100 bp; Rice et al. 2008). Phylogenetic analysis of the *V. carvalhoae* plastid genome fully resolved it in a clade sister to *V. pubescens* (Figure 3). This evolutionary relationship is congruent to a recent multilocus phylogeny among *Vasconcellea* species (Tineo et al. 2020). Pairwise genetic distances of the plastid genomes of *V. carvalhoae* and *V. pubescens* are 0.00063%. This confirms their sistership already established by the genetic divergence for the ITS region (0.36%; Tineo et al. 2020).

**Figure 3.**
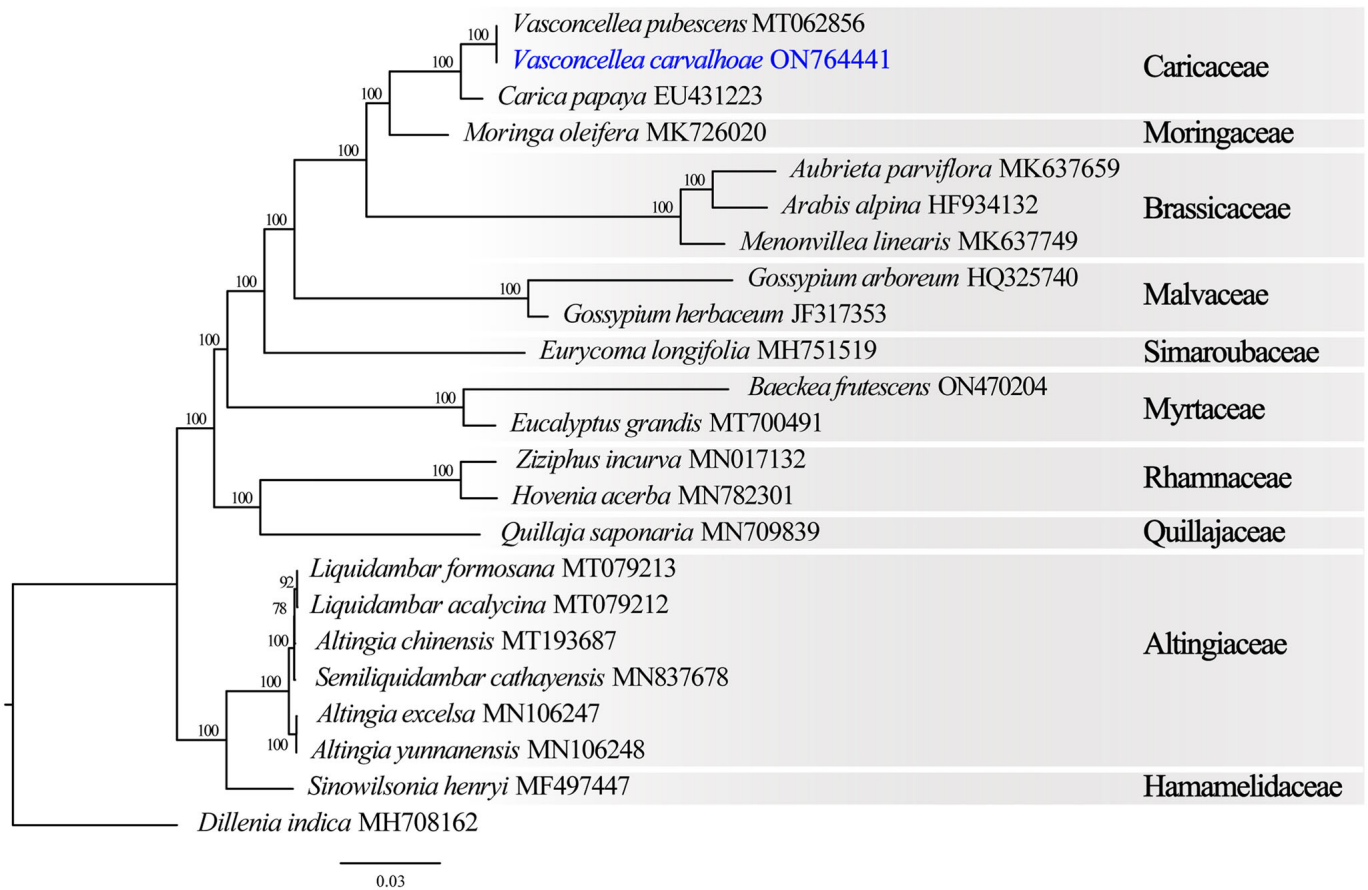
Maximum-likelihood phylogram of *Vasconcellea carvalhoae* (ON764441) and related families. Numbers along branches are RaxML bootstrap supports based on 1000 replicates. The legend below represents the scale for nucleotide substitutions.

**Table 1.** Characteristics of plastid genome among species of Caricaceae.

Characteristics	*Vasconcellea carvalhoae* (ON764441)	*Vasconcellea pubescens* (MT062856)	*Carica papaya* (NC010323)
Size (base pair, bp)	158,723	158712	160,100
LSC length (bp)	88,002	87,991	88,749
SSC length (bp)	17,841	17,841	18,701
IR length (bp)	26,440	26,440	26,325
Number of genes	130^a^	131	131
Protein-coding genes	20	20	20
Protein-coding genes	82	82	82
GC content (%)	37	37	37
tRNA genes	37	37	37
rRNA genes	8	8	8
Protein coding part (CDS) (%bp)	49.16	48.03	49.11
References	This study	Lin et al. (2020)	Lin et al. (2020)

a Lacking the infA pseudogene.

## Supplementary Material

Supplemental Material

Supplemental Material

## Data Availability

The genome sequence data that support the findings of this study are openly available in GenBank of NCBI at https://www.ncbi.nlm.nih.gov/ under the accession no. ON764441. The associated BioProject, BioSample, and SRA numbers are PRJNA851104, SAMN29211141, and SRR20115278, respectively.
