# Supplementary figures and images for: Analysis of the complete plastidial genome of the newly highland papaya Vasconcellea carvalhoae (Caricaceae) from Peru
Source: Mitochondrial DNA B Resour. 2022 Oct 27;7(10):1882–6. doi: 10.1080/23802359.2022.2135407 (PMC9621235; doi:10.1080/23802359.2022.2135407)

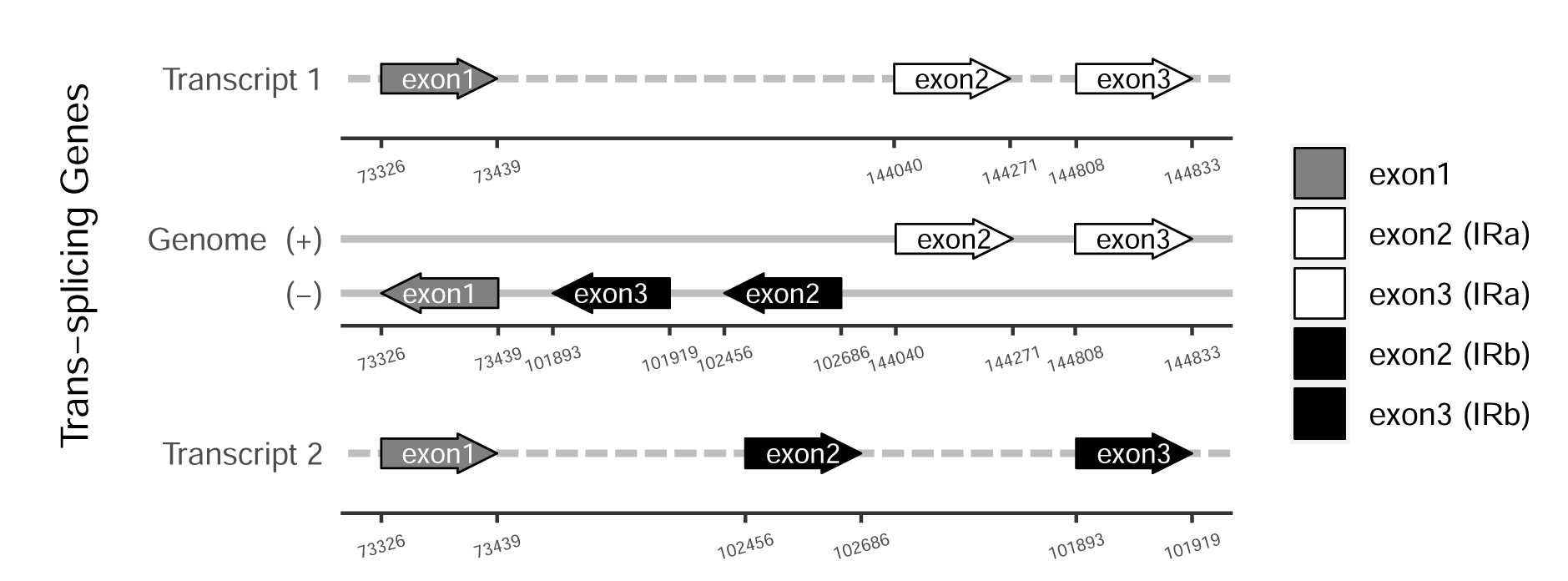

Supplement: Supplemental Material [file TMDN_A_2135407_SM5470.jpg]

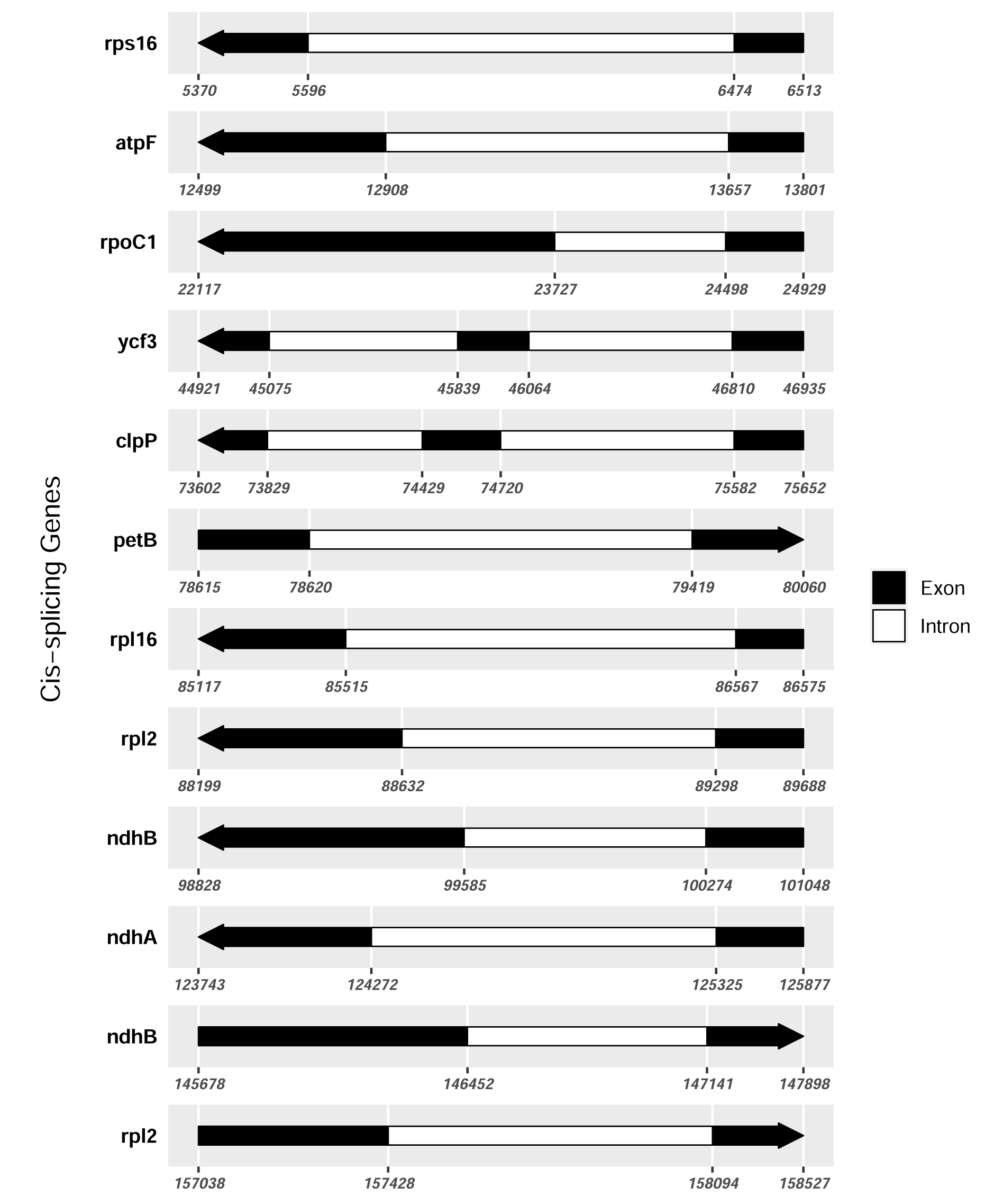

Supplement: Supplemental Material [file TMDN_A_2135407_SM5469.jpg]
